# Multivariate linkage analysis using the electrophysiological phenotypes in the COGA alcoholism data

**DOI:** 10.1186/1471-2156-6-S1-S118

**Published:** 2005-12-30

**Authors:** Heping Zhang, Xiaoyun Zhong, Yuanqing Ye

**Affiliations:** 1 Department of Epidemiology and Public Health, Yale University School of Medicine, New Haven, Connecticut, CT 06520-8034 USA; 2 Jiangxi Normal University, Nanchang, Jiangxi Province, China

## Abstract

Multivariate linkage analysis using several correlated traits may provide greater statistical power to detect susceptibility genes in loci whose effects are too small to be detected in univariate analysis. In this analysis, we apply a new approach and perform a linkage analysis of several electrophysiological phenotypes of the Collaborative Study on the Genetics of Alcoholism data of the Genetic Analysis Workshop 14. Our approach is based on a variance-component model to map candidate genes using repeated or longitudinal measurements. It can take into account covariate effects and time-dependent genetic effects in general pedigree data. We compare our results with the ones obtained by SOLAR using single measurement data. Our multivariate linkage analysis found linkage evidence on two regions on chromosome 4: around marker *GABRB1 *at 51.4 cM and marker *FABP2 *at 116.8 cM (unadjusted *p-*value = 0.00006).

## Background

The Collaborative Study on the Genetics of Alcoholism (COGA) is a large, multisite genetic study to identify susceptibility genes for alcohol dependence and related phenotypes. COGA data include information from the visual oddball experiment and the eyes closed resting electroencephalogram (EEG) dataset. The four fields beginning with ttth contain data extracted from the target case of the visual oddball experiment for four electrode placements. The extracted measures correspond to the late time window, which is set at 300 to 700 ms following stimulus presentation (bounding the visual P3 event), and the theta band power (3 to 7 Hz). The ttth1 measures have yielded a strong linkage signal on chromosome 7 [1]. The fields beginning with ttdt contain data similar to the ttth variables except that they are based on the delta band power (1 to 2.5 Hz). The fields beginning with ntth contain data extracted from the non-target case of the visual oddball experiment for four electrode placements. The extracted measures correspond to the early time window, which is set at 100 to 300 ms following the stimulus presentation, and the theta band power (3 to 7 Hz). The field labelled with ecb21 contains data extracted from the eyes closed resting EEG experiment. This measurement corresponds to the first component of a trilinear singular value decomposition of the Beta2 band (16.5 to 20 Hz) bipolar electrode data. These data have shown strong linkage on chromosome 4 and strong linkage disequilibrium (LD) with GABA-A gene single-nucleotide polymorphisms (SNPs) on chromosome 4 [2,3]. The data also include the age, labelled ERP Age, at which the electrophysiological data were collected.

Multivariate linkage analysis using several correlated traits may provide greater statistical power to detect susceptibility genes in loci whose effects are too small to be detected in univariate analysis. In this report, we analyzed the COGA data using an extension of the variance components models for repeated measurements, and considered simultaneously several of the electrophysiological phenotypes.

## Methods

We assume independence between pedigrees, and consider one pedigree to describe our model. Let *y *= (*y*_11_,...,*y*_1*m*_,...,*y*_*n*1_,...,*y*_*nm*_) be a vector of *m *multivariate trait values for *n *members of the pedigree. The *i*^th ^family member has *m *trait values observed at the age of *t*_*i*_, *i *= 1,...,*n*. Consider the model, for *i *= 1,...,*n *and *j *= 1,...,*m*,

*y*_*ij*_(*t*_*i*_) = *f*(*X*_*i*_,*t*_*i*_) + *s*(*t*_*i*_)*γ*_*i*1 _+ *γ*_*i*2 _+ *e*_*ij*_(*t*_*i*_),     (1)

where *f*(*X*_*i*_,*t*_*i*_) is a function of the fixed covariate effects *X*_*i *_and time *t*_*i*_, *s*(*t*_*i*_) a simple parametric function to accommodate time variant genetic effects, *γ*_*i*1 _the random effect for a major gene, *γ*_*i*2 _the random effect for the cumulative effect of the residual genes, and *e*_*ij*_(*t*_*i*_) the measurement error. We assume that *γ*_*i*1_, *γ*_*i*2_, and *e*_*ij *_are independent, although *e*_*ij*_(*t*_*i*_), *j *= 1,...,*m*, has a within-subject correlation structure. It follows:

cov(*y*_*ij*_(*t*_*i*_),*y*_*lk*_(*t*_*l*_)) = *s*(*t*_*i*_)*s*(*t*_*l*_)cov(*γ*_*i*1_,*γ*_*l*1_) + cov(*γ*_*i*2_,*γ*_*l*2_) + *δ*_(*i *= *l*)_*σ*_*jk*_(*t*_*i*_,*t*_*l*_),

where *σ*_*jk*_(*t*_*i*_,*t*_*l*_) is the covariance function for *e*_*ij*_(*t*_*i*_) and *e*_*lk*_(*t*_*l*_) and *δ*_(*i *= *l*) _is the identity indicator which is 1 when *i *= *l *and 0 otherwise. In addition, the covariances of *γ*_*i*1 _and *γ*_*i*2 _can be partitioned into additive and dominant variances as follows:



and



where *k*_*j *_represents the *k *coefficient of Cotterman [4] for the probability of members *i *and *l *sharing *j *alleles identically by decent (IBD) at the locus of interest, *φ *and *τ *are respectively the expected kinship coefficient and the expected probability of sharing 2 alleles IBD over the residual components of the genome,  and  are the additive and dominant genetic variances at the locus of interest, respectively, and  and  are the total additive and dominant genetic variances over the residual components of the genome, respectively. The *π*_*il*_, *k*_2,*il*_, *φ*_*il *_and *τ*_*il *_can be obtained using the SOLAR software program [5]. Because the dominant effects are usually too small, we do not consider them in this analysis. A restricted maximum likelihood approach is used to estimate parameters. A likelihood ratio test is used to test the null hypothesis that the genetic variance due to the quantitative trait locus (QTL) equals zero (no linkage). Two times the log likelihood ratio yields a test statistic that is asymptotically distributed as a mixture of *χ*^2 ^distributions [6].

The dataset includes a total of 143 nuclear and multigenerational families with 1,614 individuals. We chose those genotyped individuals with no missing electrophysiological phenotypes and ages. This yields 140 families with a total of 819 individuals. We focus our analysis on ecb21 and ttth1 and ttth2 electrophysiological phenotypes that result in a total of 2,457 measurements for the 819 individuals. We noted that from a scatter plot of each electrophysiological phenotype versus the age at which the data were collected, there was a roughly quadratic trend of the phenotype over age. Therefore, in Equation (1), we incorporated age at which the electrophysiological data were collected and its square as covariates. We also incorporated sex as a covariate and included some dummy variables as covariates to allow different intercepts for the individual phenotypes. We also performed the analysis with or without smoking status as a covariate. We considered two forms of *s*(*t*): constant and linear functions of the age. For the environmental covariance function *σ*_*jk*_(*t*_*i*_,*t*_*l*_), we assumed the same environmental variance for every phenotype (a standardization can be performed prior to the linkage analysis if this assumption is violated) and we considered either the same environmental covariance between any two phenotypes or all different environmental covariances between any two phenotypes.

## Results

We first used the SOLAR software program [4] to analyze each of the electrophysiological phenotypes. We computed the IBD information using SOLAR for two-point linkage analysis. The two-point linkage results from SOLAR show that, for ttth1 data, marker *D7S1804 *at 156.4 and *D7S509 *at 163.7 on chromosome 7 both have LOD scores around 3.5 and *p*-values reaching 0.00003, *D7S1796 *at 120 cM has a LOD score of 2.4 and a *p*-value of 0.0003, and *D7S794 *at 177.9 cM has a LOD score of 2.1 and a *p*-value of 0.0009. Consistent with existing analyses, individual ttth1 phenotypes produced strong linkage signals on chromosome 7. The results from SOLAR also showed that, for ecb21 data, marker *D4S2382 *at 43.3 cM on chromosome 4 has a *p*-value of 0.006 (LOD score = 1.4), *GABRB1 *at 51.4 cM on chromosome 4 has a *p*-value of 0.006 (LOD score = 1.4), and *FABP2 *at 116.8 cM on chromosome 4 has a *p*-value of 0.002 (LOD score = 1.7).

**Figure 1:**
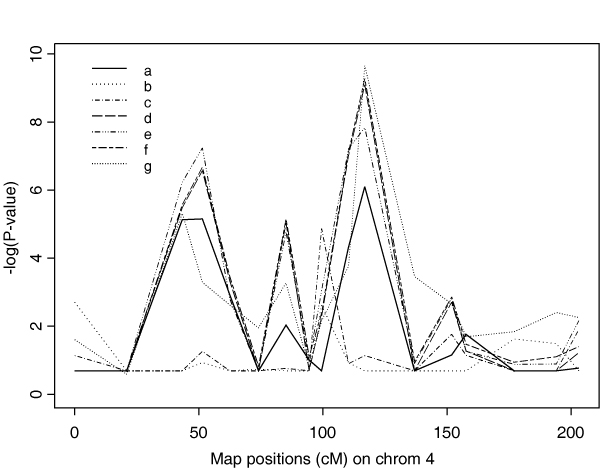
**Two-point linkage analysis. **The y-axis shows the negative of the natural logarithm of *p*-values. Curves a, b, and c are from SOLAR for ecb21, ttth1, and ttth2 data, respectively. Curve d is from our model with a constant *s*(*t*) using both ecb21 and ttth1. Curves e and f are for ecb21, ttth1 and ttth2 together, where both assume *s*(*t*) as a constant function; e assumes the same environmental covariance between any two phenotypes and f assumes different environmental covariance between any two phenotypes. Curve g is for ecb21, ttth1, and ttth2 together, assuming *s*(*t*) as a linear function and different environmental covariance between any two phenotypes.

Inclusion of smoking status as a covariate did not lead to a notable change in the test statistics. Thus, we present only the results without adjusting for smoking. Figure 1 gives the two-point linkage analysis results over chromosome 4. In the plot, the x-axis plots the map positions (in centimorgans) of the markers on the chromosome. The y-axis plots the negative of the natural logarithm of *p*-values. In the figure, curve a shows the linkage analysis results from SOLAR for ecb21 data, curve b shows the linkage analysis results from SOLAR for ttth1 data, and curve c shows the linkage analysis results from SOLAR for ttth2 data. Curve d shows the linkage analysis results from our model considering *s*(*t*) as a constant function for using both ecb21 and ttth1 data. Curve e shows the combined linkage analysis results for using ecb21, ttth1, and ttth2 from our model considering *s*(*t*) as a constant function and assuming the same environmental covariance between any two phenotypes. Curve f shows the combined linkage analysis results for using ecb21, ttth1, and ttth2 from our model considering *s*(*t*) as a constant function and assuming different environmental covariance between any two phenotypes. Curve g shows the combined linkage analysis results for using ecb21, ttth1, and ttth2 from our model considering *s*(*t*) as a linear function and assuming different environmental covariance between any two phenotypes. Because we do not have theoretical proofs for the asymptotic distributions of the test statistics, we computed the power from the simulated critical values instead of the asymptotic values. From curves a, b, and c, the univariate linkage analysis exhibits the largest value of -log(*p*-value), 6.10, (LOD score = 1.7) on curve a for ecb21 at marker *FABP2 *at 116.8 cM.

As we can see from Figure 1, curve d reaches a -log(*p*-value) of 9.08 (LOD score = 2.9) at marker *FABP2 *at 116.8 cM on chromosome 4. And, at the same marker, curve e has a value of 7.83 (LOD score = 2.4), curve f has a value of 9.27 (LOD score = 3.0) and curve g has a value of 9.65 (LOD score = 3.9). Our multivariate linkage analysis led to peaks around these two regions, marker *GABRB1 *at 51.4 cM and marker *FABP2 *at 116.8 cM, while the evidence of linkage around the two regions has been enhanced. The marker *GABRB1 *at 51.4 cM on chromosome 4 has already been identified before to be associated with alcoholism [1,2]. Considering *s*(*t*) as one constant parameter different for each phenotype deserves further investigation. Our multivariate linkage analysis did not find any significant evidence of linkage on chromosome 7. Therefore the results are not reported here.

## Discussion

In this analysis, we conducted a simultaneous linkage analysis of multivariate phenotypes. We identified some candidate markers that were identified before using some single phenotypes such as marker *GABRB1 *at 51.4 cM on chromosome 4, but also some markers that were not suggested before such as marker *FABP2 *at 116.8 cM on chromosome 4. It is also important to note that the power can also be compromised by a multivariate analysis if only one of the phenotypes contains strong linkage. For example, for chromosome 7, univariate linkage analysis for ttth1 phenotypes revealed very strong linkage signals around 156.4 cM on chromosome 7 with *p*-values reaching 0.00003, but our multivariate linkage analysis considering ttth1 and ecb21 together or considering ttth1, ttth2, and ecb21 together did not find any significant region on chromosome 7 with linkage, which may be due to the noise introduced by the phenotypes that do not have linkage signals on the chromosome. In other words, a multivariate analysis is most effective when linkage evidence to the individual phenotypes is not strong.

## Abbreviations

COGA: Collaborative Study on the Genetics of Alcoholism

EEG: Electroencephalogram

IBD: Identity by descent

LD: Linkage disequilibrium

QTL: Quantitative trait loci

SNP: Single-nucleotide polymorphism

## Authors' contributions

HZ conceived of the study, participated in the design of the study, drafted the manuscript, revised it critically for intellectual content, and gave final approval of the version to be published. XZ participated in the design of the study, performed the statistical analysis, and drafted the manuscript. YY participated in the manuscript preparation, particularly in the initial data processing. All authors read and approved the final manuscript.
